# ADHD in children and adolescents in Germany. Results of the cross-sectional KiGGS Wave 2 study and trends

**DOI:** 10.17886/RKI-GBE-2018-085

**Published:** 2018-09-19

**Authors:** Kristin Göbel, Franz Baumgarten, Benjamin Kuntz, Heike Hölling, Robert Schlack

**Affiliations:** Robert Koch Institute, Berlin, Department of Epidemiology and Health Monitoring

**Keywords:** MENTAL HEALTH, ADHD, PREVALENCE AND TIME TREND, HEALTH MONITORING, KIGGS

## Abstract

Attention deficit/hyperactivity disorder (ADHD) is one of the most common mental disorders in childhood and adolescence and is associated with functional, psychosocial and cognitive impairment. As part of the second wave of the German Health Interview and Examination Survey for Children and Adolescents (2014-2017), parents of children and adolescents aged between 3 and 17 years reported whether their child was diagnosed with ADHD by a physician or psychologist. Overall, 4.4% of children and adolescents have been diagnosed with ADHD in Germany. In comparison, the KiGGS baseline study (2003-2006) showed a reduction of lifetime ADHD diagnoses of almost one percentage point over a period of ten years. The reduction of parent-reported ADHD diagnoses primarily occurred among 3- to 8-year old children and boys. The results are discussed in terms of health promotion and the introduction of health care measures.

## Introduction

Attention deficit/hyperactivity disorder (ADHD), with its three core symptoms of inattentiveness, hyperactivity (motor unrest) and impulsivity, is one of the most common mental disorders in childhood and adolescence [1-3]. ADHD is estimated to have a worldwide prevalence of approximately 5%; this has remained relatively stable over the last few decades [1, 4, 5].

According to the diagnostic criteria used by the classification systems (International Statistical Classification of Diseases and Related Health Problems, 10th revision, ICD-10; Diagnostic and Statistical Manual of Mental Disorders, 5th Edition, DSM-5) and diagnostic guidelines (e.g. the ADHD working group of physicians in child and adolescent medicine [6]), a diagnosis of ADHD requires that core symptoms persist beyond developmentally inappropriate levels for at least six months, are pervasive across settings (e.g. school and home) and associated with substantial functional psychosocial impairment [7, 8]. Boys are diagnosed more frequently with ADHD than girls [9]. Approximately, 60% to 70% of patients with ADHD in childhood show a persistence of symptoms into adulthood [1, 10]. ADHD is commonly diagnosed among children of primary-school age [3] as symptoms become progressively more evident and cause greater impairment due to the increasing external demands placed upon children in school (e.g. maintaining attention, remaining seated) [1].

Comorbidities are highly prevalent among children and adolescents with ADHD and vary between 60% and 80% depending on the study [11, 12]. The most common comorbid mental disorders are oppositional defiant disorder (ODD), conduct disorder (CD), depression, anxiety, and learning disabilities [7, 12]. Furthermore, the symptoms of ADHD among children and adolescents are associated with a reduced subjective health-related quality of life [13, 14], increased accident-proneness [15], decreased educational attainment [16], increased disruption of family functioning [17], conflict-ridden relationships with peers [16], and social stigmatisation [18]. Moreover, alongside the significant functional impairment and the broad impact of ADHD on individuals and families, it is likely to incur a high level of health-care related costs [19, 20]. Consequently, it also has implications for health policy.

**KiGGS Wave 2**

Second follow-up to the German Health Interview and Examination Survey for Children and Adolescents

**Data owner:** Robert Koch Institute

**Aim:** Providing reliable information on health status, health-related behaviour, living conditions, protective and risk factors, and health care among children, adolescents and young adults living in Germany, with the possibility of trend and longitudinal analyses

**Study design:** Combined cross-sectional and cohort study

**Cross-sectional study in KiGGS Wave 2**

**Age range:** 0-17 years

**Population:** Children and adolescents with permanent residence in Germany

**Sampling:** Samples from official residency registries - randomly selected children and adolescents from the 167 cities and municipalities covered by the KiGGS baseline study

**Sample size:** 15,023 participants

**KiGGS cohort study in KiGGS Wave 2**

**Age range:** 10-31 years

**Sampling:** Re-invitation of everyone who took part in the KiGGS baseline study and who was willing to participate in a follow-up

**Sample size:** 10,853 participants

**KiGGS survey waves**

► KiGGS baseline study (2003-2006), examination and interview survey
► KiGGS Wave 1 (2009-2012), interview survey
► KiGGS Wave 2 (2014-2017), examination and interview survey

More information is available at www.kiggs-studie.de/english

This study presents lifetime prevalences of parent-reported ADHD diagnoses from the second wave of the German Health Interview and Examination Survey for Children and Adolescents (KiGGS Wave 2, 2014-2017). Furthermore, it also describes time trends for a period of ten years by comparing data with the KiGGS baseline study (2003-2006).

## Indicator

The German Health Interview and Examination Survey for Children and Adolescents (KiGGS) is part of the health monitoring system established at the Robert Koch Institute. KiGGS includes repeated cross-sectional surveys of children and adolescents aged between 0 and 17 years (KiGGS cross-sectional study). Both the KiGGS baseline study (2003-2006) and KiGGS Wave 2 (2014-2017) were conducted as a combined examination and interview survey. A detailed description of the methodology used in KiGGS Wave 2 can be found in New data for action. Data collection for KiGGS Wave 2 has been completed in issue S3/2017 as well as KiGGS Wave 2 cross-sectional study – participant acquisition, response rates and representativeness in issue 1/2018 of the Journal of Health Monitoring [21, 22].

The lifetime prevalence of ADHD was assessed for children and adolescents aged 3 to 17 years using parent-reported ADHD diagnoses given by a physician or a psychologist (see [23]). The results for the current lifetime prevalence are based on data from 13,270 children and adolescents (6,671 girls, 6,599 boys) between 3 and 17 years of age from KiGGS Wave 2. Data from 13,487 children and adolescents (6,736 girls, 6,751 boys) from the KiGGS baseline study were used for comparison to analyse time trends. Prevalences of ADHD diagnoses are presented stratified by gender, age and socioeconomic status (SES, [24]).

The analyses were carried out using a weighting factor that corrected for deviations within the sample from the population structure with regard to age in years, gender, federal state, German citizenship and the parents’ level of education [25]. Results report lifetime prevalences stratified by gender, age, and SES with 95% confidence intervals (95% CI). The p-values calculated for the analysis of time trends are based on age-standardised prevalences (population on 31 December 2015). Differences were examined using univariate logistic regression. A statistically significant difference between groups is assumed to have been demonstrated where p-values are less than 0.05.

## Results and discussion

Overall, 4.4% of children and adolescents between 3 and 17 years of age showed a parent-reported lifetime diagnosis of ADHD given by a physician or psychologist for KiGGS Wave 2 (2014-2017) (Table 1). For the KiGGS baseline study, the prevalence of age-adjusted lifetime ADHD diagnoses was 5.3%. In contrast, a significant reduction in the prevalence of ADHD diagnoses of 0.9 percentage points (corresponding to about 17 % compared to the baseline value) was identified from the data collected for KiGGS Wave 2. A gender comparison showed a significant reduction in the number of ADHD diagnoses among boys. No significant difference was observed for girls between both waves.

In comparison to the KiGGS baseline study, the results suggest that the diagnostic gap between girls and boys may be closing, however, the data show that boys are still diagnosed with ADHD twice as often as girls. Moreover, the time trend demonstrates that lifetime ADHD diagnoses significantly dropped among 3- to 5-year-olds and 6- to 8-year-olds. No valid findings about gender specific differences within age groups can be made due to the small sample size within each group.

**Figure 1:**
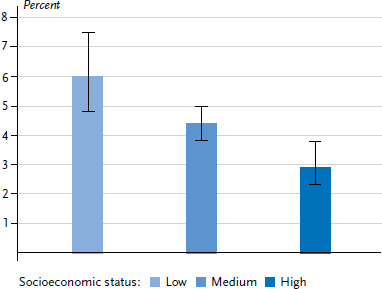
Prevalences for parent-reported ADHD diagnoses* for 3- to 17-year-olds according to socioeconomic status (n=6,671 girls, n=6,599 boys) Source: KiGGS Wave 2 (2014-2017) * ADHD = Attention deficit/hyperactivity disorder

Children and adolescents living in socioeconomically disadvantaged families are more frequently diagnosed with ADHD compared to their peers from high SES families (Figure 1). Previous results of the KiGGS baseline study and KiGGS Wave 1 confirm distinctive differences between the prevalence of ADHD diagnoses which are to the disadvantage of lower SES households [3].

Over the last decade, a continuous increase in media reports and research, as well as the reported rise in the diagnostic prevalence of ADHD, have driven a broad debate involving society, health policy, medical and psychological health care professionals, and service providers in the health system (see e.g. [26]).

This debate has resulted in the initiation of several measures related to health care policy and provision. One example is the directive issued by the Federal Joint Committee (G-BA) for the modification of drug policies aimed at a more restrictive prescription of psychostimulants (i.e. methylphenidate) in the case of children and adolescents with ADHD [27]. The directive reflects the concern about the rate of prescriptions provided for stimulants as this increased over several years. Subsequently, medical guidelines for the diagnostics and therapy of ADHD have also been adapted (see [6]) and the Scientific Medical Societies in Germany (AWMF) has recently (June 2018) published a new version of its guidelines [28].

It cannot be ruled out that these changes may have led to the introduction of a more restrictive diagnostic practice in the case of ADHD. This assumption is supported by the recent reduction in the lifetime prevalence of ADHD among the youngest age groups, particularly as these were at the focus of the debate and have continued to be so. Other initiatives to promote children’s health at the federal level have also been introduced. These include the German government’s strategy for the promotion of children’s health, the promotion of the national centre for early support, which began in 2007, and the medical check-up (U10) put in place in 2006. However, the impact that they might have had on the prevalence of ADHD diagnoses remains open.

The results show that boys are still diagnosed twice as often with ADHD as girls. Boys are referred for treatment for ADHD more often and earlier as their behaviour tends to be more disruptive (i.e. more hyperactive or impulsive) than girls [29]. A previous study found that ADHD is almost equally distributed between girls and boys when a higher level of diagnostic recognition is placed on the inattentive subtype and the less overt symptoms of ADHD which are more commonly found among girls [30].

Conclusions about the accuracy of ADHD diagnoses, whether these follow the guidelines, or the severity of the disorder cannot be drawn from the parent-reported ADHD diagnoses collected for the KiGGS study. Additionally, the question as to whether the parents’ response behaviour towards ADHD diagnoses might have been influenced by the changing public and professional perception over the last ten years remains open.

Overall, the study found a significant reduction in the diagnostic prevalence of about one percentage point over a period of ten years. Hence, it cannot be ruled out that this reduction might be a consequence of a more restrictive diagnostic practice for ADHD. This explanation is currently supported by routine data gained from statutory health insurers that report a small reduction in ADHD diagnoses [31].

## Key statements

- 4.4% of children and adolescents between 3 and 17 years of age had received a parent-reported lifetime diagnosis of ADHD from a physician or psychologist at KiGGS Wave 2 (2014-2017).
- In contrast to the KiGGS baseline study (2003-2006), KiGGS Wave 2 showed a significant reduction in the prevalence of ADHD diagnoses of about one percentage point.
- A reduction of parent-reported ADHD diagnoses was identified among boys and 3- to 8-year old children.

## Figures and Tables

**Table 1:** Prevalences for parent-reported ADHD* diagnoses according to gender and age from the KiGGS baseline study (n=6,736 girls, n=6,751 boys) and KiGGS Wave 2 (n=6,671 girls, n=6,599 boys) Source: KiGGS baseline study (2003-2006), KiGGS Wave 2 (2014-2017) * ADHD = Attention deficit/hyperactivity disorder

	KiGGS baseline study		KiGGS Wave 2	
	%	(95 % CI)	%	(95 % CI)
**Girls**	**1.9**	**(1.5-2.4)**	**2.3**	**(1.9-2.8)**
**Boys**	**8.5**	**(7.7-9.5)**	**6.5**	**(5.7-7.3)**
**Age group**				
3-5 Years	1.5	(1.0-2.3)	0.2	(0.1-0.7)
6-8 Years	3.8	(3.1-4.7)	2.1	(1.5-2.9)
9-11 Years	7.6	(6.4-8.9)	6.1	(4.9-7.5)
12-14 Years	6.7	(5.6-7.9)	6.4	(5.3-7.6)
15-17 Years	6.4	(5.4-7.7)	6.9	(5.8-8.2)
**Total (girls and boys)**	**5.3**	**(4.8-5.8)**	**4.4**	**(3.9-4.9)**

CI=confidence interval

## References

[1] Banaschewski T Becker K Döpfner M (2017) Attention-Deficit/Hyperactivity Disorder. Dtsch Arztebl Int 114(9):149-159 28351467 10.3238/arztebl.2017.0149 PMC5378980

[2] Polanczyk G De Lima MS Horta BL (2007) The worldwide prevalence of ADHD: a systematic review and metaregression analysis. Am J Psychiatry 164(6):942-948 17541055 10.1176/ajp.2007.164.6.942

[3] Schlack R Mauz E Hebebrand J (2014) Hat die Häufigkeit elternberichteter Diagnosen einer Aufmerksamkeitsdefizit-/Hyperaktivitätsstörung (ADHS) in Deutschland zwischen 2003–2006 und 2009–2012 zugenommen? Bundesgesundheitsbl 57(7):820-829 10.1007/s00103-014-1983-7 24950831

[4] Polanczyk GV Willcutt EG Salum GA (2014) ADHD prevalence estimates across three decades: an updated systematic review and meta-regression analysis. Int J Epidemiol 43(2):434-442 24464188 10.1093/ije/dyt261 PMC4817588

[5] Safer DJ (2018) Is ADHD really increasing in youth? Journal of attention disorders 22(2):107-115 26084287 10.1177/1087054715586571

[6] AG ADHS e.V. (2014) Leitlinie der Arbeitsgemeinschaft ADHS der Kinder- und Jugendärzte e.V. Aktualisierte Fassung Januar 2007. Mit Update des Kapitels „Medikamentöse Therapie“ März 2014. https://www.ag-adhs.de/files/Leitlinie2014mr.pdf (As at 10.07.2018)

[7] Döpfner M Frölich J Lehmkuhl G (2013) Aufmerksamkeitsdefizit-/Hyperaktivitätsstörung (ADHS). Hogrefe Verlag, Göttingen

[8] Tarver J Daley D Sayal K (2014) Attention deficit hyperactivity disorder (ADHD): an updated review of the essential facts. Child Care Health Dev 40(6):762-774 24725022 10.1111/cch.12139

[9] Huss M Hölling H Kurth BM (2008) How often are German children and adolescents diagnosed with ADHD? Prevalence based on the judgment of health care professionals: results of the German health and examination survey (KiGGS). Eur Child Adolesc Psychiatry 17 Suppl 1:52-58 10.1007/s00787-008-1006-z 19132304

[10] Groß S Figge C Matthies S (2015) ADHS im Erwachsenenalter. Der Nervenarzt 86(9):1171-1180 26341837 10.1007/s00115-015-4328-3

[11] Kadesjö B Gillberg C (2001) The comorbidity of ADHD in the general population of Swedish school-age children. J Child Psychol Psychiatry 42(4):487-492 11383964

[12] Larson K Russ SA Kahn RS (2011) Patterns of comorbidity, functioning, and service use for US children with ADHD, 2007. Pediatrics 127(3):462-470 21300675 10.1542/peds.2010-0165 PMC3065146

[13] Hölling H Schlack R Dippelhofer A (2008) Personale, familiäre und soziale Schutzfaktoren und gesundheitsbezogene Lebensqualität chronisch kranker Kinder und Jugendlicher. Bundesgesundheitsbl 51(6):606 10.1007/s00103-008-0537-2 18465100

[14] Klassen AF Miller A Fine S (2004) Health-related quality of life in children and adolescents who have a diagnosis of attention-deficit/hyperactivity disorder. Pediatrics 114(5):e541-547 15520087 10.1542/peds.2004-0844

[15] Ruiz-Goikoetxea M Cortese S Aznárez-Sanado M (2018) Risk of unintentional injuries in children and adolescents with ADHD and the impact of ADHD medications: a systematic review and meta-analysis. Neurosci Biobehav Rev 84:63-71 29162520 10.1016/j.neubiorev.2017.11.007

[16] Schulte-Körne G (2016) Psychische Störungen bei Kindern und Jugendlichen im schulischen Umfeld. Dtsch Arztebl Int 113(11):183-190 27118666 10.3238/arztebl.2016.0183 PMC4850518

[17] Wymbs BT Pelham WE Jr Molina BS (2008) Rate and predictors of divorce among parents of youths with ADHD. J Consult Clin Psychol 76(5):735 18837591 10.1037/a0012719 PMC2631569

[18] Lebowitz MS (2016) Stigmatization of ADHD: A Developmental Review. J Atten Disord 20(3):199-205 23407279 10.1177/1087054712475211

[19] Erskine H Moffitt TE Copeland W (2015) A heavy burden on young minds: the global burden of mental and substance use disorders in children and youth. Psychol Med 45(7):1551-1563 25534496 10.1017/S0033291714002888 PMC5922255

[20] Schöffski O Sohn S Happich M (2008) Die gesamtgesellschaftliche Belastung durch die hyperkinetische Störung (HKS) bzw. Aufmerksamkeitsdefizit-/Hyperaktivitätsstörung (ADHS). Gesundheitswesen 70(07):398-403 18729028 10.1055/s-0028-1082049

[21] Hoffmann R Lange M Butschalowsky H (2018) KiGGS Wave 2 cross-sectional study – participant acquisition, response rates and representativeness. Journal of Health Monitoring 3(1):78-91. https://edoc.rki.de/handle/176904/5637 (As at 10.07.2018) 10.17886/RKI-GBE-2018-032 PMC8848911 35586176

[22] Mauz E Gößwald A Kamtsiuris P (2017) New data for action. Data collection for KiGGS Wave 2 has been completed. Journal of Health Monitoring 2(S3):2-27. https://edoc.rki.de/handle/176904/2812 (As at 10.07.2018) 10.17886/RKI-GBE-2017-105 PMC10291840 37377941

[23] Schlack R Hölling H Kurth BM (2007) Die Prävalenz der Aufmerksamkeitsdefizit-/Hyperaktivitätsstörung (ADHS) bei Kindern und Jugendlichen in Deutschland. Bundesgesundheitsbl 50(5-6):827-835. https://edoc.rki.de/handle/176904/431 (As at 10.07.2018) 10.1007/s00103-007-0246-2 17514469

[24] Lampert T Hoebel J Kuntz B (2018) Socioeconomic status and subjective social status measurement in KiGGS Wave 2. Journal of Health Monitoring 3(1):108-125. https://edoc.rki.de/handle/176904/5639 (As at 15.03.2018) 10.17886/RKI-GBE-2018-033 PMC8848848 35586179

[25] Forschungsdatenzentren der Statistischen Ämter des Bundes und der Länder (2017) Mikrozensus,. 2013, eigene Berechnungen. www.forschungsdatenzentrum.de/bestand/mikrozensus (As at 20.11.2017)

[26] AG ADHS e.V. (2009) Gutachten des Sachverständigenrates und Stellungnahme des zentralen adhs-netzes zur Begutachtung der Entwicklung im Gesundheitswesen. http://www.adhs-deutschland.de/Home/Unser-Angebot/Leseecke/Leseecke-Politik/Gutachten-des-Sachverstaendigenrates-zur-Begutachung-der-Entwicklung-im-Gesundheitswesen.aspx (As at 10.08.2018)

[27] Gemeinsamer Bundesausschuss (G-BA) (2010) Zum Schutz von Kindern und Jugendlichen – Verordnung von Stimulantien nur in bestimmten Ausnahmefällen - Beschluss: Arzneimittel-Richtlinie/Anlage III Nummer 44 (Stimulantien) https://www.g-ba.de/informationen/beschluesse/1185/ (As at 10.07.2018)

[28] Arbeitsgemeinschaft der Wissenschaftlichen Medizinischen Fachgesellschaften (AWMF) (2018) S3-Leitlinie zur Diagnostik und Behandlung von Kindern, Jugendlichen und Erwachsenen mit ADHS. https://www.awmf.org/leitlinien/detail/ll/028-045.html (As at 11.07.2018)

[29] Skogli EW Teicher MH Andersen PN (2013) ADHD in girls and boys–gender differences in co-existing symptoms and executive function measures. BMC psychiatry 13(1):298 24206839 10.1186/1471-244X-13-298 PMC3827008

[30] Biederman J Kwon A Aleardi M (2005) Absence of gender effects on attention deficit hyperactivity disorder: findings in nonreferred subjects. Am J Psychiatry 162(6):1083-1089 15930056 10.1176/appi.ajp.162.6.1083

[31] Grobe TG (2017) Regionale Unterschiede von ADHS-Diagnoseraten in Krankenkassendaten 2005 bis 2015. Bundesgesundheitsbl 60(12):1336-1345 10.1007/s00103-017-2640-8 29052741

